# Snakebite epidemiology and health-seeking behavior in Akonolinga health district, Cameroon: Cross-sectional study

**DOI:** 10.1371/journal.pntd.0008334

**Published:** 2020-06-25

**Authors:** Gabriel Alcoba, Manon Chabloz, Justin Eyong, Franck Wanda, Carlos Ochoa, Eric Comte, Armand Nkwescheu, François Chappuis

**Affiliations:** 1 Division of Tropical and Humanitarian Medicine, Geneva University Hospitals, Geneva, Switzerland; 2 Médecins Sans Frontières-Doctors Without Borders (MSF), Geneva, Switzerland; 3 Faculty of Medicine, University of Geneva, Geneva, Switzerland; 4 Centre International de Recherche, d'Enseignement et de Soins en Milieu Tropical (CIRES), Akonolinga, Cameroon; 5 Institute for Environmental Sciences, GeoHealth group, and Institute of Global Health, University of Geneva, Geneva, Switzerland; 6 Cameroon Society of Epidemiology (CaSE), Yaoundé, Cameroon; 7 Faculty of Medicine and Biomedical Science, University of Yaoundé 1, Yaoundé, Cameroon; Monash University, AUSTRALIA

## Abstract

**Background:**

Snakebite envenoming causes 81,000–138,000 annual human deaths and pain, terror, or disability in 4.5–5.4 million victims. Accurate community-based epidemiological data is scarce. Our objective was to assess snakebite incidence, mortality, and health-seeking behavior, in an affected health district of Cameroon.

**Methods:**

We conducted a cross-sectional multicluster household survey in Akonolinga health district, Centre Region, Cameroon, from October to December 2016. Using probability-proportional-to-size, 20 villages were randomly selected, then, all inhabited households were systematically selected. Annual incidence and adjusted odds-ratio for predictors were estimated.

**Findings:**

Among the 9,924 participants, 66 suffered a snakebite during the past year: the resulting incidence is 665 (95%CI: 519–841) per 100,000 inhabitants per year. Victims were aged 5-75y (median: 34y), 53% were male and 57% farmer-cultivators. Two children died (case-fatality rate: 3%); 39 (59%) presented severity signs, including 2 (3%) neurotoxic syndromes, 20 (30%) systemic digestive syndromes, and 17 (26%) severe cytotoxic syndromes. Non-severe cases included 20 (30%) mild cytotoxic syndromes and 7 (11%) dry bites. Only two victims (3%) received antivenom. 59 (89%) used family traditional practices, 25 (38%) traditional healers, and 31 (47%) consulted health facilities. Median delays to these three care-options were 5, 45, and 60 minutes, respectively. Traditional treatments included incisions (n = 57; 86%), tourniquets (n = 51; 77%) and black-stones (n = 44; 67%). The two last procedures were also used in health facilities (n = 18). Consulting traditional healers was associated with severity (adjusted-OR: 19.6 (2.5–156), p = 0.005) and complications (aOR: 17.3, 2.4–123, p = 0.004). Long-term disabilities were subjective psychological trauma (n = 47; 71%), finger amputation (n = 1; 2%), ankylosis (n = 1; 2%) and chronic pain (n = 1; 2%).

**Conclusions:**

We observed alarming levels of snakebite incidence, mortality, antivenom scarcity, and use of traditional medicine. It could represent several thousands of victims at national level. We suggested conducting a country-wide study, and improving antivenom supply, first-aid training, for traditional healers and health professionals.

## Author summary

On a global scale, snakebite envenoming causes more than 100,000 human deaths annually, as well as pain, terror, or serious disabilities in about 5 million victims, as reported by the World Health Organization. However, accurate data from community-based surveys are lacking, especially in Sub-Saharan Africa. Our aim was to assess the burden of snakebite in Akonolinga health district in Centre Region, Cameroon. We conducted a cross-sectional household survey in 20 randomly selected villages. **Our study shows that about 6.6 victims per 1,000 inhabitants are bitten by a snake yearly, and that 3% of those victims die**. The two victims who died in our survey were children who did not receive antivenom. Among all victims, 59% presented with severe symptoms, but only 3% received antivenom, and the majority used some traditional treatment. Consulting traditional healers was associated with clinical severity and medical complications. In summary, we found three main problems: a high burden in terms of incidence, severity and death; a lack of access to antivenoms; and the common use of potentially harmful traditional practices (causing delay and complications). This could represent tens of thousands of victims on a national level. We suggest conducting a country-wide representative study.

## Introduction

Snakebite envenoming is increasingly recognized as a major, deadly tropical disease of public health importance. Snakebite was re-included in the World Health Organization’s (WHO) list of Neglected Tropical Diseases (NTDs) in June 2017 [1,2], highlighting the lack of (*i*) epidemiological awareness, (*ii*) adequate prevention and medical training, and (*iii*) safe, affordable and polyvalent antivenoms in many regions.

WHO estimates that yearly 4.5–5.4 million people are bitten by a snake, of which 1.8–2.7 million are envenomed [3,4], leading to about 400,000 amputations or lifelong disabilities, and 81,000–138,000 deaths. Snakebite is a major hazard in many tropical countries, where human activities and animal habitat often overlap [3,4]. Human-snake contacts occur mainly during field activities, in high grass or forest walks, but also around or in houses in regions of high density of snake population, typically farming regions where grain attracts rodents that, in turn, attract snakes.

In Cameroon, 150 snake species have been identified, more than 20% of which are potentially harmful to humans [5]. The northern regions are populated with deadly carpet vipers (*Echis ocellatus*). The humid equatorial climate of central Cameroon hosts different cytotoxic and haemotoxic vipers (*Bitis gabonica*, *Bitis nasicornis* and *Atheris squamigera*), whose venoms induce local tissue destruction that may lead to necrosis and amputation, or render blood non-coagulable, resulting in hemorrhagic shock [6]. The elapids endemic to this area are neurotoxic cobras and mambas (*e*.*g*. *Naja melanoleuca* and *Dendroaspis jamesoni)*, whose venoms cause a flaccid paralysis of the cranial nerves, rapidly extending downwards to the respiratory muscles [6]. Elapids also include non-neurotoxic, cytotoxic, spitting cobras *(e*.*g*. *Naja nigricollis)*. Snakes causing more moderate and local cytotoxic symptoms include burrowing asps (*Atractaspis)*, some colubridae (*Crotaphopeltis*) and small adders (*Causus spp*.) [6].

The existing epidemiological estimations, globally [3,4], and in sub-Saharan Africa [7], are mainly hospital-based, underestimating the true incidence and the consequences of snakebite in the community. Some bites are rapidly lethal, so the victims die before reaching any health facility; out-of-hospital traditional treatment is also frequent, when antivenoms are not easily available, factors that all contribute to under-reporting. Out-of-hospital treatment is undergone by up to 80% of the victims, either practiced by themselves, by community members, or by traditional healers [8]. Tourniquets are often used to try to limit the spread of the venom, despite the dangerous risk of ischemia of the distal limb. WHO and some national guidelines have banned the practice [8–10]. The application of the very popular black-stone has been proven ineffective because insufficiently specific to venom proteins, although the absorbing capacity of some types of these “snake stones” has been observed [11,12]. Regarding any benefit of herbal traditional treatments, extensive research suggested encouraging local effects of many plants [13–17] but never when they are used in the raw manner in which they are used in communities [18].

Many snakebite survivors suffer life-long disabilities. Amputations described in a few studies in Sub-Saharan Africa [8–12] are among the many physical consequences of snakebite. Psychological stress and its impact on working capacities are not to be neglected. Up to 10% of victims stop working after the bite [19], and snakebites have cost over 290,000 Years of Life Lost and over 29,000 Years of Life with Disability in 16 countries of West Africa. Antivenom treatment has been proven cost-effective in West-Africa [20] and is the only recommended treatment to this day.

Sub-Saharan Africa is, along with Southern Asia, one of the most snakebite-affected regions in the world. In 2015, the Cameroonian Ministry of Public Health started reporting weekly snakebite hospital admissions at the health district level. This strong initiative is among the first, globally, at this frequency. In 2018 the Cameroon Society of Epidemiology reported more than 3,000 yearly victims of snakebite and a 2.5% lethality [21]. However, this estimate is based on hospital-provided data, excluding victims who could not consult or who died before reaching a health facility. An international workshop in Yaoundé in November 2015 [22] brought together medical and traditional practitioners, as well as specialists in herpetology, snakebite clinical management, antivenoms, and public health authorities, to provide medical training to health professionals. This workshop highlighted clinical training and inclusive inter-disciplinary coordination as key priorities.

We aimed to measure the annual incidence of snakebite at the community level in Akonolinga, a health district of the Centre Region, Cameroon, to assess its clinical severity, mortality, its long term-complications and the victims’ health-seeking behavior.

## Methods

The health district of Akonolinga expands over a territory of about 3,400 km2 and holds a population of 105,789 inhabitants. Its climate is tropical and its populations work mostly in agriculture, for exportation and for their own subsistence, according to the Ministry of Agriculture.

This population-based epidemiological study was a two-stage cross-sectional household survey: the first level of selection was a random selection of clusters after dividing villages into equal clusters, proportionally to the size of the population of each village; the second level of selection was a complete (exhaustive) survey of all the members of the cluster. Villages were weighed by PPS (population proportional to size) and randomly selected by the online tool Research Randomizer. In order to reach a statistically adequate sample size, 20 villages were selected, representing a population of 14,067 inhabitants, representing 13.3% of the total district official population (105,789 inhabitants). Data collection took place between October and December 2016.

Door-to-door investigation was conducted by two mobile teams of two numerators each, guided by a local community health worker (ReCo, *relai communautaire*) with previous experience in community-based health campaigns (*e*.*g*. vaccination or mosquito-net distribution). The data collectors were one doctor, two nurses, and one physiotherapist, all Cameroonians. All of them had already participated in community door-to-door work (mosquito net distribution, population census, vaccination campaigns). They underwent a specific training for this project with one of the Swiss investigators, in July 2016 in Akonolinga, which included theoretical training on the clinical manifestations of snakebite envenoming, and role-playing exercises for the proper execution of the questionnaire. The questions were created by Cameroonian and Swiss investigators together. Their syntax was adapted to the field with the help of the data collectors in July 2016, to ensure optimal comprehension by the victims.

The number of permanent inhabitants and the presence of snakebite victims were asked for in each household. To provide an annual incidence and to limit recall (memory) bias, victims were included only if bitten between 1 July 2015 and 30 June 2016. An adult member of the family was asked to answer the questionnaire on behalf of minor (under 21 years old in Cameroon) or deceased snakebite victims. All the victims encountered agreed to participate in the study. Information forms were distributed and written informed consent was obtained from every victim.

Questionnaires were filled on electronic tablets (ACER Iconia one 10) using the KoBoCollect software (kobotoolbox.org /Harvard Humanitarian Initiative). Questions to victims covered demographical characteristics, snakebite context, snake characteristics (including identification in a snake photo album), health-seeking pathway (care-givers, treatments and delays), and symptoms before and after treatments, chronic disabilities, and prevention methods used.

The language used between the data collectors and snakebite victims was a derivate of the Ewondo language. In both collecting teams, one of the investigators was fluent in this language, and the other one had good comprehension of it. The questionnaire itself was in French, which all of the four investigators spoke fluently. The investigators inserted the answers given by the victims on the questionnaire in the tablet.

Data were edited, encoded, and analyzed using STATA 14.0 (StataCorp LLC). The annual incidence was calculated with the real population count, using a count of each household composition as the denominator, and expressed with 95% confidence intervals (CI). Odds ratio (OR) with CI were calculated to analyze individual risk factors. Multivariate regression analysis was used to search independent risk factors, expressed through adjusted OR (aOR).

Three different types of caregivers were considered. The victims themselves, their family members, or any other member of the village other than a health professional or a traditional healer, were all designated as “self-/family-treatment”. The other caregivers were traditional healers and health care professionals.

Envenoming types were identified using pre- and post-treatment symptoms. Attempts to identify the classic neurotoxic, cytotoxic and haemotoxic syndromes proposed by the WHO [6] were limited by the victim’s description of frequent non-specific and few specific clinical signs or symptoms. We used a practical classification of severity (where all systemic syndromes, neuro- and haemotoxic, as well as severe cytotoxic syndromes are considered severe) based on the WHO classic syndromes. Victims describing systemic symptoms (neurologic and non-specific hypotension, abdominal pain, diarrhea, vomiting) were all considered severe cases. Victims describing only pain and/or local swelling were considered non-systemic cases and were sub-classified as dry bite (no swelling), mild cytotoxic cases (limited swelling) or severe cytotoxic cases (swelling progressing over one major proximal joint such as wrist or ankle).

Given the short delay between the bites and (any) treatment, symptoms appearing after the first treatment and fully resolved at the moment of the study were considered both as an effect of the envenoming (as described above) and as a potential acute complication of the received treatments. Chronic disabilities were defined as permanent physical or mental conditions remaining after the bite. Psychological trauma was defined by conscious avoidance of specific places or activities related to the snakebite, and/or frequent nightmares about the event. Based on the DSM-V criteria for Post-traumatic Stress Disorder, we considered that there could be a possible psychological trauma resulting from the snakebite when the victim responded positively to either one of the following questions: Do you consciously avoid specific places or activities related to the snakebite? Or: Do you have frequent nightmares about the event? We did not use specialized scales such as the Center for Epidemiologic Studies Depression Scale Revised (CESD-R) for depression, or the Post-traumatic Stress Disorder Symptom Scale Self Report (PSS-SR) for Post-Traumatic Stress Disorder, which require further specialized training.

Ethical approval and study authorizations were obtained in Cameroon, from the *Comité National d’Ethique de la Recherche pour la Santé Humaine* (CNERSH), and the Ministry of Public Health, and in Switzerland, from the *Commission Cantonale d’Ethique de la Recherche scientifique* (CCER).

## Results

### Incidence and mortality

This population-based cross-sectional study in 20 random villages enrolled 1,649 households (9,924 inhabitants) in Akonolinga health district, Centre Region, Cameroon. In total, 66 snakebites and two deaths were reported, indicating an annual incidence of 665 per 100,000 inhabitants and a case-fatality rate of 3%.

### Victim characteristics

The typical victim was a 34-year-old (median age, interquartile range 16–53) male (53%) whose main occupation was farmer/fieldworker (57%; **Table 1**). Most bites occurred between June and November, the main rainy season (n = 47, 71%), with the highest monthly incidence in July (n = 15, 23%), between 6 a.m. and 6 p.m. (n = 43, 65%), while the victims were in the fields/plantations (n = 26, 40%). One victim (2%) was bitten in her sleep. Fifty bites (76%) involved the lower limb. Only two victims (3%) gave a coherent description of the snake genus responsible for the bite (name, color, and picture identification), *Atractaspis sp*. in both cases. Incoherent descriptions of the snake were given by 45 victims (68%) and 28 did not see the snake at all (29%).

**Table 1:** Snakebite in Akonolinga health district: victim characteristics, health-seeking pathways and negative outcomes.

Socio-demographic and medical characteristics n = 66 snakebite victims (out of 9,924 participants)	Number (%)
**Sex**	
Male	35 (53)
Female	31 (47)
**Age**	
Median (IQR), years	34 (16–53)
Range, years	5–75
**Occupation**	
Field-plantation worker/farmer	38 (58)
Student/pupil	19 (29)
Other professionals (lawyers, physicians, teachers)	3 (4)
Traders	2 (3)
Fishermen	2 (3)
Unemployed	2 (3)
**Type of healthcare provider**	
Self- or family-administered treatment	59 (89)
Traditional healer	25 (38)
Hospital/health center	31 (47)
**Negative outcomes**	
Acute complication	32 (49)
Chronic disability	49 (74)
Deaths	2 (3)

### Bite demographics

**Fig 1:**
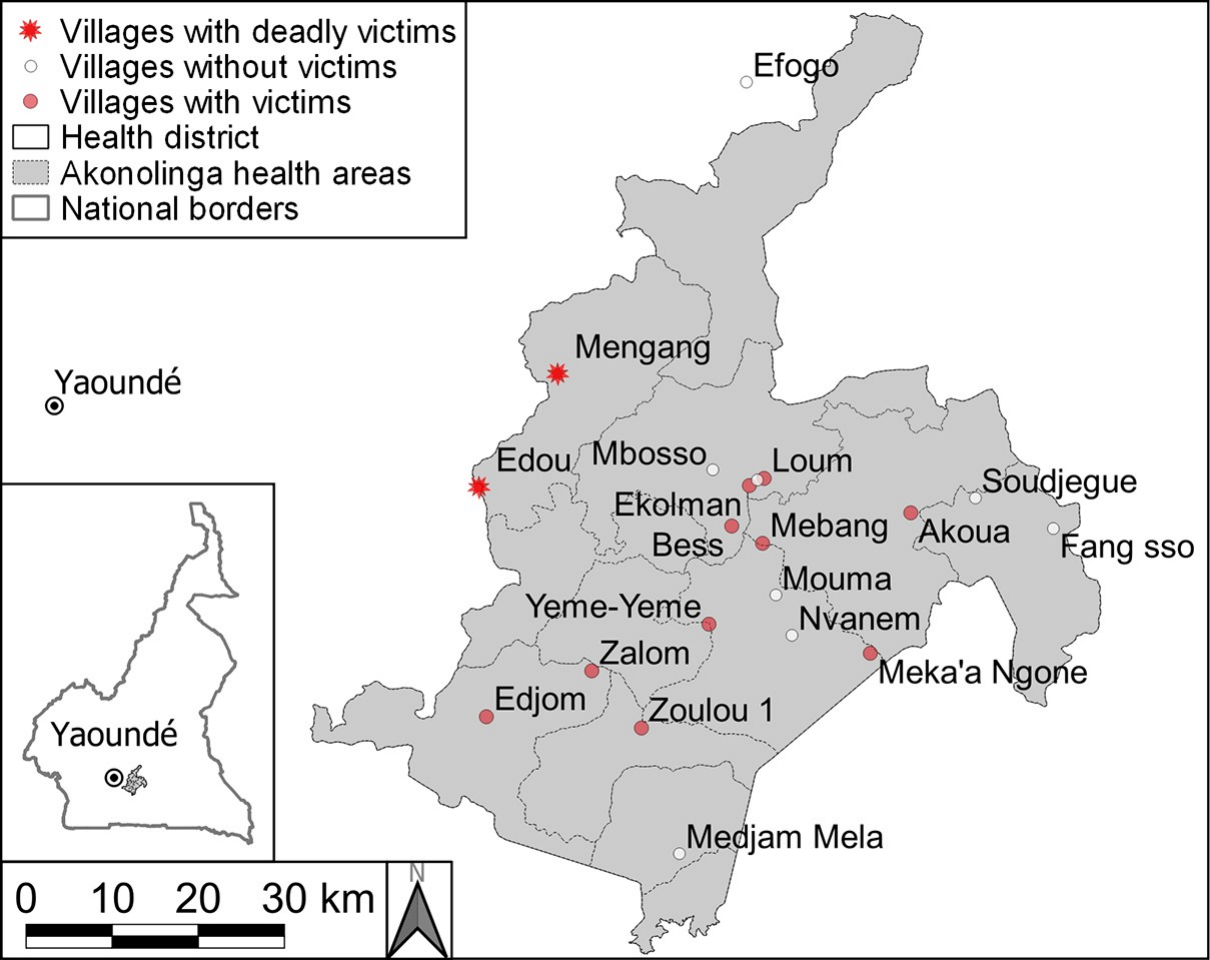
Map of 20 randomly selected villages in Akonolinga health district Centre region without (white dots) and with snakebite victims (red dots), and snakebite deaths (red stars).

Regarding geospatial distribution, **Fig 1** shows the randomly selected villages where the survey was performed, and those where snakebite victims were found. Given the relatively low numbers it is difficult to calculate village-specific attack rates. Edou and Mengang were the villages where the two deaths occurred. Out of the 20 villages, 12 hosted at least one snakebite victim. Edou alone reported over 20% of the total yearly victims (13 out of 66), followed by Zalom (n = 11), Mengang (n = 10), Mebang (n = 9), Akoua (n = 7), Edjom (n = 6), Loum (n = 3), Yeme-yeme and Zoulou (n = 2 each), Bess, Ekolman, and Meka N’gone (n = 1 each). No snakebites were reported in the other eight villages. The map in **Fig 1** does not show a clear distribution pattern for snakebite occurrences throughout the district, but there was a tendency to higher frequency and lethality in the Western villages, at a short distance of the main river, and in the deep forest area.

### Envenoming severity

**Fig 2:**
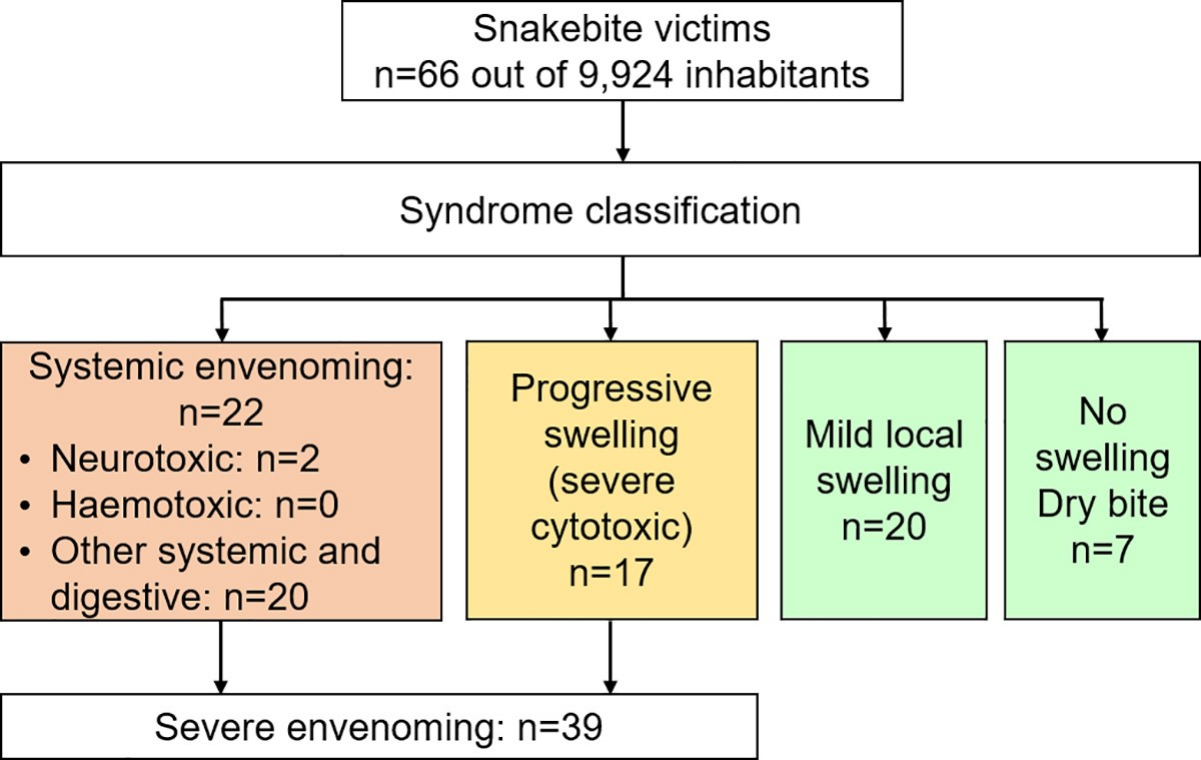
Flowchart and classification of all snakebite envenoming syndromes.

Two children of five and seven years old died over the study period, one from neurotoxic and one from digestive symptoms (intense vomiting) unfortunately without more details, but apparently leading to systemic shock and organ failure. Although severe envenoming occurred more often when the victim was over 5 years old (p = 0.033), the risk of mortality was much higher in children ≤ 5 years old (OR 31, CI 1.4–694, p = 0.030). Location at the time of the bite, gender of the victim or seasonality were not identified as predictors of negative outcomes. **Fig 2** summarizes the presentation of all snakebite victims classified per severity. The neurotoxic syndrome found in two victims (3%) was significantly associated with death (p<0.001, OR 63, CI 2–1,895). The most common syndromes were systemic-digestive (n = 20, 30%), mild cytotoxic (n = 20, 30%), severe cytotoxic syndrome (n = 17, 26%), and finally dry bites (n = 7, 11%).

### Treatments

Fifty-nine victims (89%) received initial self/family-treatment, within a median delay of 5 minutes (interquartile range [IQR]: 5–15min). Twenty-five victims (38%) consulted a traditional healer, within a median delay of 45 minutes (IQR 20–60 minutes, max.17h). Thirty-one victims (47%) received treatment by a health care professional, within a median delay of 45 minutes (IQR 22–90 minutes, max. 2h) if they went directly, but 60 minutes (IQR 40–120 minutes, max.7 days) if they went after seeking family/traditional treatments. Most (93%, n = 25) of those with a prehospital treatment took more than 30 minutes to get to the facility, compared to only 50% (n = 2) among those who went directly to the health facility (p = 0.018).

All treatments received are listed in **Table 2**. A total of 14 different treatments were performed by the victims themselves or their families. Tourniquets (n = 48, 80%), incisions (n = 32, 53%) and black-stones (n = 29, 48%) were the commonest, but other, less known treatments were also recorded, such as urine application (n = 15, 23%), snake bile ingestion (n = 1, 2%), water (n = 2, 3%) or oil (n = 1, 2%) massages, and songs and prayers (n = 1, 2%). Herbal decoctions, whether ingested or applied on the bite site, were the main treatments performed by traditional healers (n = 22, 88%). Incisions were performed by all caregivers, health professionals included. In eleven cases (36%) black stones were applied in health facilities. Among the 39 severely envenomed victims for whom antivenom was indicated, 18 (46%) presented to a health center and only 2 (5%) received antivenom. These two antivenom treatments were delivered by the same health center, but unfortunately, the two children who died could not access any antivenom. Injectable medications received in health centers were, according to the victims, corticoids, antibiotics or anti-tetanic serum.

**Table 2:** Snakebite treatments in Akonolinga health district.

Snakebite treatments	Victim or family (%) n = 59	Traditional healer (%) n = 25	Health center (%) n = 31
**Conventional practices**			
Anti-venom	0 (0)	0 (0)	2 (6.5)
Intra-venous medication (any)	6 (10)	0 (0)	31 (100)
Oral medication	3 (5)	0 (0)	18 (58)
Surgical debridement	0 (0)	0 (0)	1 (3)
Wound bandaging	1 (2)	0 (0)	6 (19)
Wound cleaning	1 (2)	0 (0)	4 (13)
**Traditional practices**			
Tourniquet	48 (80)	3 (12)	0 (0)
Incision	32 (53)	18 (72)	7 (23)
Black stone	29 (48)	4 (16)	11 (36)
Urine (application or ingestion)	15 (23)	0 (0)	0 (0)
Herbs (application or ingestion)	10 (15)	22 (88)	0 (0)
Suction	3 (5)	1 (4)	0 (0)
Hot water massage	2 (3)	0 (0)	0 (0)
Prayers/chants	1 (2)	0 (0)	0 (0)
Snake bile (ingestion)	1 (2)	0 (0)	0 (0)
Olive oil (application)	1 (2)	0 (0)	0 (0)

### Acute complications

Almost half of the victims (n = 32, 48%) developed new symptoms after receiving their first treatment, which could either be collateral effects of the treatments, or effects of the progression of the envenoming. These symptoms were mainly cytotoxic: progressive swelling (56%), suspected compartment syndrome (6%), generalized swelling (allergic or cytotoxic; 3%), and necrosis (3%), as well as wound infections (6%). Neurological complications were also mentioned: numbness of the bitten limb, decreased sight and ptosis (each at 6%) as well as respiratory paralysis (bradypnea and dyspnea), and hallucinations (maybe due to hypoxia; each at 3%). Pain and fatigue were also common non-specific consequences described (6% each). As previously mentioned, the cause of these complications could not be identified (treatments or snake venom). For example, local necrosis or blistering could be from the direct effect of the venom, but could also result from the tightness of the tourniquet, from herbal applications or from traditional incisions. Still, none of the complications could be significantly associated with any specific treatment.

### Chronic disabilities

Within the timeframe of this study, chronic disabilities persisted in 49 victims (74%): subjective psychological trauma (n = 47, 96%), amputation of two distal phalanges (n = 1, 2%) and ankylosis of the remaining phalange (n = 1, 2%), and chronic recurring pain in the bitten limb, in the form of intense tingling (n = 1, 2%). None of the disabilities could be significantly associated with any specific treatment.

### Health-seeking pathways as risk factors

Health-seeking pathways and different determinants of the snakebite context were analyzed to identify possible association with severe envenoming, acute complications or chronic disabilities (**Table 3**). Health facility consultation, whether as an exclusive pathway (“health facility ONLY”) or combined with other caregivers (“health facility”) was not significantly associated either with severe envenoming (p = 0.15 and p = 0.997 respectively) or with acute complications (p = 0.16 and p = 0.33 respectively). In contrast, traditional healer consultation when combined with other caregivers (“traditional healer”), was associated with severity of envenoming (adjusted multivariate odd ratio, aOR = 19.6 [CI 2.5–156], p = 0.005) and also with acute complications (aOR = 17.3 [2.4–123], p = 0.004). We could not show the same association when it was the only caregiver consulted (“traditional healer ONLY”). Finally, self- or family-administration of traditional practices did not appear as associated with excess risk of severity or complications; it was even significantly associated with reduced severity (aOR = 0.11 [0.02–063], p = 0.013) and reduced complications (aOR = 0.92 [0.015–0.57], p = 0.011), when used as an exclusive pathway. None of the six pathways could be significantly associated with the occurrence of chronic disabilities.

**Table 3:** Envenoming severity and acute complications according to health-seeking pathways, with univariate and multivariate adjusted odds ratios (aOR, adjusted for potential confounders: Age, sex, profession, village, habitat, time, season).

Health-seeking pathways (n = 66)		Severe envenoming			Acute complications		
	Total N	N (%)	Crude OR (95%CI)	Adjusted OR (95%CI), *P-value*	N (%)	Crude OR (95%CI)	Adjusted OR (95%CI), *P-value*
**Health facility**	31	18 (58)	0.92 (0.35–2.5)	1.0 (0.19–5.2), 0.997	15 (48)	1.1 (0.43–2.97)	2.3 (0.42–13), 0.33
**Health facility ONLY**	4	2 (50)	0.68 (0.09–5.12)	0.12 (.006–2.3), 0.15	1 (25)	0.31 (0.03–3.2)	0.12 (0.005–2.4), 0.16
**Self-family care**	59	34 (58)	0.54 (0.097–9.0	0.87 (0.12–6.5), 0.89	14 (70)	1.38 (0.28–6.7)	1.8 (0.23–13), 0.59
**Self-family care** **ONLY**	15	5 (33)	0.25 (0.07–0.84)	0.11 (0.02–0.6), **0.013**	3 (20)	0.18 (0.04–0.70)	0.92 (0.015–0.57), **0.011**
**Traditional healer**	25	20 (80)	4.6 (1.5–15)	19.6 (2.46–156.1), **0.005**	18 (100)	4.5 (1.5–13)	17.3 (2.4–123), **0.004**
**Traditional healer ONLY**	3	3 (100)	NA*, p = 0.14	NA*, p = 0.88	2 (66)	2.1 (0.17–24)	3.2 (0.17–62), 0.44

*OR and aOR not measurable for this small sample

### Snakebite prevention

Finally, regarding snakebite preventive measures, fifty-one victims (77%) reported applying at least one preventive measure to avoid snakebites. The most common measure (47%) was covering their ankles when outdoors, using long pants, boots, or two pairs of socks. Other methods were cutting long grass around the house (15%), placing repulsive plants around the house (14%), receiving a traditional preventive treatment (12%), everyday vigilance in outdoor activities (9%), ingesting a herbal decoction (5%), sleeping under a mosquito net (5%), interrupting the activity performed when the bite occurred (3%), having a black-stone in the pocket (2%), having a domestic animal (2%), and avoiding going out at night (2%). No further association is described.

## Discussion

### High snakebite incidence

The community incidence of snakebite found in this study in the Centre Region of Cameroon was higher than that found previously in other Sub-Saharan African countries such as Benin (440/100,000 inhabitants/year [23]) and Nigeria (497/100,000 [24]). On the contrary, the case fatality ratio (3%) was lower than what was found in Benin (5.9% [23]) or Nigeria (12.2% [24]). The fatalities in this study occurred in small children (5 and 7 years old), showing, as elsewhere, that young age is a risk factor for deadly envenoming, due to the higher ratio of venom dose and body weight.

### Lack of antivenom

Regarding snakebite treatments, we observed a very low rate of antivenom use even for severe cases, but a very frequent use of traditional treatments by all three types of caregivers, including health professionals who are not supposed to use incisions, tourniquets and other dangerous practices. This resulted in the absence of a clear benefit in survival or complications in patients who consulted health-facilities. The excessive use of traditional treatments in healthcare facilities points to their lack of antivenom supply and lack of updated skills about modern case management of snakebite, as observed in previous qualitative work [22] during a national training and workshop in Yaoundé in 2015. Indeed, only one health center in this study stored antivenom, fulfilling only 5% (2 of 39 severely envenomed victims) of the needs. One of the young girls who died of envenoming had been brought to a health-center, but no antivenom was available. Currently few antivenoms are available or truly polyvalent. The choice of antivenom is not easy at central level, and should be targeted per regions for the moment, which is also complex. Next-generation monoclonal antibodies (mAbs) mixtures and PLA2 inhibitors (varespladib) are promising [25,26], but have not undergone clinical trials yet.

The strong association of traditional healer consultation with envenoming severity and complications probably simply means that severely envenomed patients consulted traditional healers more often than patient with non-severe bites; not necessarily that traditional healers were on the causal pathway of these complications. Indeed, traditional healers are probably the only trusted option in many remote villages. Therefore, acute complications following traditional healer treatment are most probably the product of the progression of envenoming, the wasted time, as well as the potentially harmful treatment. The improvement of snakebite envenoming outcomes could be achieved by supplying health centers with antivenom, supporting community awareness, and improving coordination between health centers and traditional healers to allow quick patient referral in severe cases.

Despite progress since the 2015 workshop in Yaoundé and improved central notification in 2016–2019 there is probably still a lack of notification of snakebite cases in health centers of the Akonolinga health district. Our non-exhaustive study of the health district identified 17 snakebite victims bitten between June and December 2015 who consulted a health center. However, over the entire year of 2015 and over the entire health district, only 15 snakebite cases were reported by the health centers, showing a rough 50% under-reporting of the health center consultation. Under-reporting was also described by a hospital and community-based survey in the same region [27]. Improving case notification could be a lever for health centers in providing themselves with adequate antivenom supply according to their needs.

### High population awareness

Further findings in this study suggest a high level of community awareness for snakebites. First, non-severe envenoming was strongly associated with self-treatment at home and did not lead to referrals nor significant complication rates, suggesting that the population is skilled in recognizing envenoming severity, and knows to seek further help when needed. This matches our initial hypothesis that severe cases are being referred to traditional healers while non-severe cases are managed in communities. Secondly, the delays between care-givers were surprisingly short, especially to health centers (60 minutes compared to a median delay of over 7 hours for hospital admission in South Africa [28]). Thirdly, prevention strategies were common (77%) and some of the most frequently used were those recommended by experts and WHO (covering feet and ankles, cutting high grass around houses).

However, some improvements are still needed on a community level as well. We did not specify if the application of the preventive measures had started before or only after the bite, but it is probable that the bites themselves are major motivational factors for the application of prevention strategies. Primary prevention should be encouraged to avoid bites in the first place. Furthermore, none of the first-aid treatments applied by the communities were among the ones promoted by the WHO, such as immobilizing the bitten limb and removing constrictive clothing. Therefore, snakebite prevention and first aid could be the purpose of community awareness campaigns. However, we believe that the lack of antivenom is the main bottleneck for the improvement of snakebite envenoming outcomes, and of trust towards official health facilities.

### Hidden socio-economic impact

Further research is needed to determine the extent of reported psychological trauma, which subjectively burdens 71% of the victims according to our non-specialized questionnaire, as well as the economic impact of the victims who interrupted their activity since the bite (n = 3, 5%). As reported in our introduction, both have already been shown to burden the lives of snakebite victims and the societies most affected by snakebite [29,30].

### Study limitations

Recall bias from the victims was a potential obstacle, similar to all cross-sectional studies using questionnaires on past events. However, the study period was relatively short, and a snakebite is a traumatic event, therefore it is easier to remember than mild diseases (common cold, or diarrhea). Some recall bias may have impacted snake identification, estimation of delays between care-options, and description of the symptoms presented. Limited medical knowledge from the victims, and some lack of precise syndrome classification could have had a slight impact as well. As mentioned in our Methods section, our evaluation of the psychological impact of snakebite was based on the DSM-V PTSD diagnosis criteria but we did not use any specialized scale, which is why we do not claim to have diagnosed snakebite victims with Post-Traumatic Stress Disorder. Rather, we suggest that there seems to be a psychological impact of snakebites on their victims, the characteristics and extent of which should benefit from a specialized evaluation.

### Conclusion

This study in Akonolinga health district found an alarming annual incidence of snakebite envenoming, and a high case fatality rate. Snakebite caused avoidable deaths, psychological and physical disabilities. Most care-providers involved in snakebite management provided traditional treatments rather than WHO-recommended first aid and treatments. The health district holds almost no stocks of antivenom, which was found in only one out of 20 health facilities. Our results show that antivenom scarcity was a greater threat to the victims than time to access to health care, which was short in our study. Therefore, antivenom supply seems to be the number one priority. The second priority would be the training of medical staff about safe and effective management of snakebite, about the severity criteria to use (and spare) antivenoms, and about anaphylaxis monitoring. Third, prevention and quick referral to the nearest health centers–currently well done in most places–are to be further encouraged in communities. Finally, as suggested through the workshops in Yaoundé with the Ministry of Public Health, epidemiological research and regular surveillance are needed to better understand seasonality and hotspots.

These four axes join the WHO Strategy for Prevention and Control of Snakebite Envenoming launched on 23 May 2019, which promoted a comprehensive package on community awareness, snakebite mapping, medical training and improved access to appropriate antivenoms.

Beyond our findings, this study was a simple, short, reliable, and inexpensive survey that inspired our decision to perform a much larger nationally representative epidemiological study on snakebites. It could also serve as a template, to be used as a simple and rapid snakebite assessment tool. It can also be used as a quick comparative pre-/post-intervention survey.

### Ethical approvals

Obtained in Cameroon, *Comité National d’Ethique de la Recherche pour la Santé Humaine* (CNERSH) on 3 October 2016, and in Switzerland, *Commission Cantonale d’Ethique de la Recherche scientifique* (CCER) on 14 February 2017, with permission to begin the study obtained on 19 July 2016.

## Supporting information

S1 Checklist STROBE statement for cross-sectional studies. (DOCX)
