# Supplementary material for: Snakebite epidemiology and health-seeking behavior in Akonolinga health district, Cameroon: Cross-sectional study
Source: PLoS Negl Trop Dis. 2020 Jun 25;14(6):e0008334. doi: 10.1371/journal.pntd.0008334 (PMC7343182; doi:10.1371/journal.pntd.0008334)
Supplement: S1 Checklist — (DOCX) [file pntd.0008334.s001.docx]

STROBE Statement—Checklist of items that should be included in reports of ***cross-sectional studies***

|  | Item No | Location in SNAKO manuscript |
| --- | --- | --- |
| **Title and abstract** | 1 | (*a*) Title |
|  |  | (*b*) Abstract, section Methods, section Results |
| Introduction | | |
| Background/rationale | 2 | Introduction, paragraph 1 |
| Objectives | 3 | Introduction, paragraph 2 |
| Methods | | |
| Study design | 4 | Methods, paragraph 3 |
| Setting | 5 | Methods, paragraph 3 |
| Participants | 6 | Methods, paragraph 1-2 |
| Variables | 7 | Methods, paragraph 3 |
| Data sources/ measurement | 8* | Methods, paragraph 2 |
| Bias | 9 | Methods, paragraph 2 |
| Study size | 10 | Methods, paragraph 1 |
| Quantitative variables | 11 | Methods, paragraph 3-5 |
| Statistical methods | 12 | (a) Methods, paragraph 5 |
|  |  | (b) Methods, paragraph 5 |
|  |  | (c) Methods, paragraph 5 |
| Results | | |
| Participants | 13* | (a) Results, paragraph 1 |
|  |  | (b) Results, paragraph 1 |
|  |  | (c) Flow diagram Figure 2 *(Figure 2: Flowchart and classification of all snakebite envenoming syndromes)* |
| Descriptive data | 14* | (a) Results, paragraph 1-3 and tables 1 and 2 |
|  |  | (b) Results, paragraph 1-3 |
| Outcome data | 15* | Results, paragraph 4-5 |
| Main results | 16 | (*a*) Results, paragraph 5-8 |
|  |  | (*b*) Results, paragraph 5-8 |
| Other analyses | 17 | Results, paragraph 9 |
| Discussion | | |
| Key results | 18 | Discussion, paragraphs 1-4 |
| Limitations | 19 | Discussion, paragraph 8 |
| Interpretation | 20 | Discussion, paragraphs 5-7 Conclusion, paragraphs 1-2 |
| Generalisability | 21 | Conclusion, paragraph 3 |
| Other information | | |
| Funding | 22 | Funding section |

*Give information separately for exposed and unexposed groups.

**Note:** An Explanation and Elaboration article discusses each checklist item and gives methodological background and published examples of transparent reporting. The STROBE checklist is best used in conjunction with this article (freely available on the Web sites of PLoS Medicine at http://www.plosmedicine.org/, Annals of Internal Medicine at http://www.annals.org/, and Epidemiology at http://www.epidem.com/). Information on the STROBE Initiative is available at www.strobe-statement.org.
